# Loss of Paid Employment up to 4 Years after Colorectal Cancer Diagnosis—A Nationwide Register-Based Study with a Population-Based Reference Group

**DOI:** 10.3390/cancers13122868

**Published:** 2021-06-08

**Authors:** Astrid de Wind, Sietske J. Tamminga, Claudia A. G. Bony, Maren Diether, Martijn Ludwig, Miranda J. Velthuis, Saskia F. A. Duijts, Angela G. E. M. de Boer

**Affiliations:** 1 Amsterdam UMC, Department of Public and Occupational Health, Coronel Institute of Occupational Health, Amsterdam Public Health research institute, University of Amsterdam, 1105 AZ Amsterdam, The Netherlands; s.tamminga1@amsterdamumc.nl (S.J.T.); cbony@deloitte.nl (C.A.G.B.); mdiether@deloitte.nl (M.D.); a.g.deboer@amsterdamumc.nl (A.G.E.M.d.B.); 2 Deloitte Consulting Netherlands, Analytics & Cognitive, 1081 LA Amsterdam, The Netherlands; maludwig@deloitte.nl; 3 Netherlands Comprehensive Cancer Organisation (IKNL), 3511 DT Utrecht, The Netherlands; m.velthuis@iknl.nl; 4 Amsterdam UMC, Department of Public and Occupational Health, Amsterdam Public Health Research Institute, Vrije Universiteit Amsterdam, 1081 BT Amsterdam, The Netherlands; s.duijts@amsterdamumc.nl

**Keywords:** colorectal cancer, cancer survivorship, return to work, unemployment, disability pension, register-based study

## Abstract

**Simple Summary:**

Previous research indicated that cancer survivors have a higher risk of loss of paid employment. This is unfortunate as work is important for cancer survivors because it contributes to financial independency and quality of life. Not much work has been done on patients diagnosed with colorectal cancer while it is one of the most common cancers in the working population. We compared a group of 12,007 colorectal cancer survivors up to four years after diagnosis with the general population. We found that colorectal cancer survivors had a 56% higher risk of loss of paid employment, mainly due to work disability. Within the group of colorectal cancer survivors, those being younger, having a higher cancer stage and receiving radiotherapy, had a higher risk of loss of paid employment. Colorectal cancer survivors at high risk of loss of paid employment may benefit from work support interventions as part of cancer survivorship care.

**Abstract:**

Cancer survivors consider work as a key aspect of cancer survivorship while previous research indicated that cancer survivors have a higher risk of unemployment. The objectives were to assess: (1) whether colorectal cancer survivors less often have paid employment at diagnosis compared to a population-based reference group, (2) whether colorectal cancer survivors with paid work have a higher risk of loss of employment up to 4 years after diagnosis compared to a population-based reference group and (3) which colorectal cancer survivors are at highest risk of loss of paid employment. In a nationwide register-based study, persons diagnosed with colorectal cancer (*N* = 12,007) as registered in the Netherlands Cancer Registry, were compared on loss of paid employment with a sex and age-matched population-based reference group (*N* = 48,028) from Statistics Netherlands. Cox regression analyses were conducted. Colorectal cancer survivors had a higher risk of loss of paid employment (HR 1.56 [1.42, 1.71]). Within the group of colorectal cancer survivors, risk of loss of paid employment was lower for older survivors (>60 vs. 45–55) (HR 0.64 [0.51, 0.81]) and higher for those with a more advanced cancer stage (IV vs. I) (HR 1.89 [1.33, 2.70]) and those receiving radiotherapy (HR 1.37 [1.15, 1.63]). Colorectal cancer survivors at high risk of loss of paid employment may benefit from work support interventions as part of cancer survivorship.

## 1. Introduction

Survival rates of colorectal cancer have increased in recent years. The overall 5-year survival rate of colorectal cancer diagnosed in the period 2011–2014 was about 65% [1]. With a yearly incidence of colorectal cancer in the working age population of approximately 5000 [1] and an overall employment rate of about 70% in the Netherlands [2], a large number of people diagnosed with colorectal cancer is working at the time of diagnosis. This number is expected to increase due to the implementation of national colorectal cancer screening in many Western countries [3], which leads to increased incidence of colorectal cancer diagnosis at an early stage in younger patients. Additionally, increasing the statutory retirement age in many Western countries may contribute to increasing numbers of colorectal cancer survivors in the working age.

Cancer survivors in general have a 1.4 higher risk of becoming unemployed [4]. This is unfortunate, as unemployment leads to worse health outcomes [5,6,7,8]. Furthermore, cancer survivors perceive work as a key aspect of cancer survivorship [9] because it provides a feeling of contributing to society [10,11] and because it is associated with better quality of life [7,12,13,14] and a better financial situation [15,16,17]. This higher risk of becoming unemployed after a cancer diagnosis is undesirable from a societal perspective as well, as it leads to high costs associated with paying out the increased social security costs.

The reason why cancer survivors are at a higher risk of unemployment involves various impeding factors at the interface between the healthcare system and the work environment, embedded in a social security system [18,19]. For example, previous research found that cancer-related factors such as adjuvant treatment and work-related factors such as type of employment contract are associated with unemployment among cancer survivors [20]. Work disability research in cancer has predominately focused on breast cancer [21,22], and was thus mainly restricted to women, while sex and cancer diagnosis specific factors may also be of importance. It would, therefore, be worthwhile to study the work-related consequences of a cancer that occurs frequently in both men and women, as this would give insight on the influence of sex and diagnosis specific factors as well. Colorectal cancer is one of the most common cancer types in men and women of working age with relatively good survival rates, and would, thus, be of interest [1].

A systematic review including eight studies on prognostic factors for return to work in colorectal cancer survivors found that adjuvant treatment, more comorbidities and higher age were negatively associated with return to work, and that extensive surgical resection, complications and a previous period of unemployment were associated with work disability [23]. Additionally, previous research found that approximately 60–89% of the colorectal cancer survivors were able to return to work, with chemotherapy and low prediagnosis socioeconomic status being barriers for return to work [24,25,26,27,28,29]. This large variation in the ability to return to work underlines the need to identify colorectal cancer survivors at high risk of loss of paid employment to support the colorectal cancer survivors who need the most help.

Identification of high risk colorectal cancer survivors is challenging because people who need help the most, do participate in research less often [30,31,32]. Due to this bias, the sociodemographic, disease-specific and work-related characteristics that determine which colorectal cancer survivors are at high risk of unemployment on a population level remain largely unknown. Consequently tailored interventions are hampered. A population-based study could be helpful for identification of high risk colorectal cancer survivors because such a study is non-selective by definition.

Therefore, the objectives of our study were to assess in a population-based study: (1) whether colorectal cancer survivors less often have paid employment at diagnosis compared to a population-based reference group, (2) whether colorectal cancer survivors with paid work have a higher risk of loss of employment up to 4 years after diagnosis compared to a population-based reference group and (3) which colorectal cancer survivors are at the highest risk of loss of paid employment.

## 2. Materials and Methods

### 2.1. Datasets and Study Sample

The present study used register data on patients diagnosed with colon or rectum cancer, including disease-specific and treatment-related factors, from the Netherlands Cancer Registry, which is managed by the Netherlands Comprehensive Cancer Organisation (IKNL) [33]. Patients were individually linked to register data on sociodemographic and work-related characteristics, income and employment status of Statistics Netherlands. The NCR contains data on all persons who have been diagnosed with cancer in the Netherlands since 1989 [1]. In addition to the individual linkage of the NCR and Statistics Netherlands, we have composed a reference sample from the general population, which was also based on register data from Statistics Netherlands, including people without a colon or rectum cancer diagnosis in the same period. Results are based on calculations by Amsterdam UMC using these non-public microdata from Statistics Netherlands [2].

**Figure 1:**
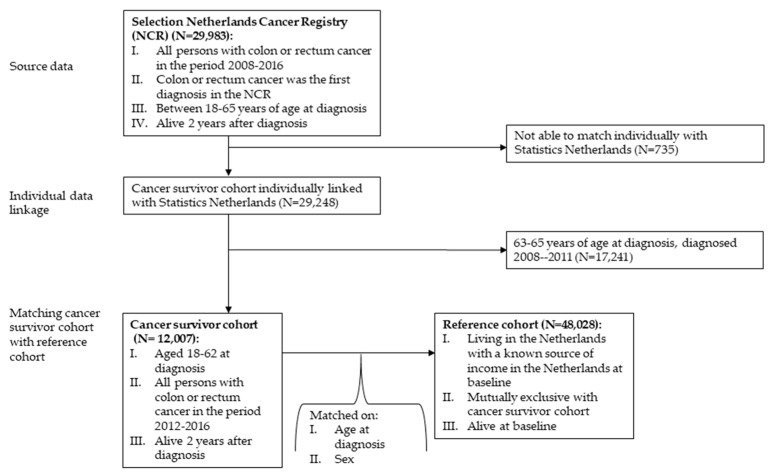
Flow of the study sample.

Inclusion criteria for the cohort of cancer survivors are the following: (i) being diagnosed with colon or rectum cancer using the International Classification of Diseases for Oncology codes C18-C20 in the period 2012–2016, (ii) colon or rectum cancer being their first cancer diagnosis, (iii) being alive two years after diagnosis and (iv) aged 18–62 years at diagnosis. We chose this age range so that all people in all follow-up years are of working age with the statutory retirement age being 66 years of age in 2018 in the Netherlands. People with colon or rectum cancer as a secondary diagnosis, i.e., metastases, were excluded. Cancer patients are only registered in the NCR when they are treated in the Netherlands, and thus, most likely, have a known source of income in the Netherlands. These inclusion and exclusion criteria resulted in a cancer survivor sample of 12,007 people (Figure 1).

From Statistics Netherlands, a population-based reference cohort was randomly selected that was comparable in terms of age and sex. For each cancer survivor, four persons were selected who: (i) were alive at baseline (2012–1016), (ii) were living in the Netherlands with a known source of income at baseline, (iii) were mutually exclusive with the cancer cohort and (iv) were exactly the same in terms of age and sex. The reference cohort was not selected on being alive two years after baseline, as the odds that someone dies in this period are negligible [2]. This resulted in a reference sample consisting of 48,028 people. The total study sample, i.e., the cancer survivor sample and reference sample together, consisted of 60,035 people (Figure 1).

### 2.2. Measures

Sociodemographic and work-related characteristics were retrieved from Statistics Netherlands. Specifically, the municipal personal records database, the employee insurance agency database, and the tax income database were used. Cancer-related characteristics were retrieved from the NCR. See Supplementary Material Table S1 for a detailed description of all measures.

#### 2.2.1. Sociodemographic Characteristics

For both subsamples, the following sociodemographic characteristics were used: age at baseline, sex (i.e., male and female) and income. We used the following age group strata: <45, 45–54, 55–59 and 60–62 years. Regarding income we distinguished between the following net income groups: <500 €, 500–20 k€, 20 k–35 k€, 35 k–50 k€ and >50 k€ yearly.

#### 2.2.2. Work-Related Characteristics

For both subsamples, the following work-related characteristics at the individual level were used: type of employment (i.e., employee and self-employed) and employment relationship (i.e., fixed and flexible working hours (e.g., temporary agency workers)).

#### 2.2.3. Cancer-Related Characteristics

For the cancer sample, the following cancer-related characteristics were used: cancer stage (i.e., I, II, III and IV) and cancer treatment (i.e., surgery, chemotherapy, radiotherapy and targeted treatment).

#### 2.2.4. Loss of Paid Employment

Loss of paid employment was conceptualized as a transition from paid employment to disability benefits, unemployment benefits and social welfare and loss of paid employment through any of these exit routes. In the Netherlands, disability benefits can be applied for when having an employment contract and being on sick leave for 2 years, or earlier in the sick leave process when recovery is considered very unlikely. Unemployment benefits can be awarded when a person loses his or her job and depends on a person’s employment history. Social welfare can be applied for when unemployment benefits are expired or when someone does not qualify for unemployment benefits and/or disability benefits. The exit routes are operationalized based on monthly information on the most important income source. Combinations of income sources (e.g., partly disability benefits and unemployment benefits) were not taken into account.

### 2.3. Statistical Analyses

Descriptive statistics of the cancer survivor sample and the reference sample were examined and reported separately for the two samples. We report descriptive statistics for age, sex, income and work-related characteristics for the month prior to diagnosis. For the cancer survivor sample, we also report on cancer-related characteristics. Furthermore, the loss of paid employment at baseline was determined and compared between the cancer survivor sample and the reference sample (first objective) and the cumulative incidence of loss of paid employment during follow-up. In accordance with disclosure guidelines of Statistics Netherlands, no counts <10 were reported.

To address the second objective, Cox regression analyses were conducted. Only cancer survivors and controls who had paid employment before diagnosis were included in these analyses. The year of colorectal cancer diagnosis of the cancer survivor was considered the baseline and the 4 years thereafter the follow-up. In the analyses, we only took into account first events, i.e., disability benefits, unemployment benefits and social welfare. Persons were censored when they were lost to follow-up (e.g., because of death or migration) before they reached one of the outcomes (i.e., disability benefits, unemployment benefits, social welfare and loss of paid employment). First, univariate Cox regression analyses were conducted with colorectal cancer diagnosis, age, sex, income, type of employment and contract type as independent variables and the different conceptualizations of loss of paid employment as dependent variables. Second, multivariate Cox regression analyses were conducted including all factors with a level of significance of *p* < 0.05 in the previous univariate analyses, except for factors with high multicollinearity (i.e., Spearman correlation coefficient ≥0.70) and factors that violated the proportional hazards assumption. This assumption is the key assumption for Cox regression analyses. It implies that the effect of a change in a covariate on the hazard rate of event occurrence is constant over time. The proportional hazards assumption was checked using statistical tests and graphical diagnostics based on Schoenfeld residuals. A plot of the Schoenfeld residuals that shows a non-random pattern against time is evidence of violation of the proportional hazards assumption [34,35]. Since we expected that a lot of hazards over time were non-proportional [36], which also appeared to be the case in exploratory analyses, the main analyses for disability benefits and loss of paid employment were performed for two separate time intervals after diagnosis based on the Dutch social security system (see Supplementary Material Table S1): up to 2 years after cancer diagnosis and 2–4 years after cancer diagnosis. For each multivariate Cox regression model the concordance index is reported, which is an indication of how well the variables included in the models predict the time to loss of paid employment.

To address the third objective, Cox regression analyses were conducted within the cancer survivor sample. The same procedures have been followed as described above. Univariate Cox regression analyses were conducted with age, sex, income, type of employment, contract type, cancer stage (I, II, III and IV), surgery (yes/no), chemotherapy (yes/no), radiotherapy (yes/no) and targeted treatment (yes/no) as independent variables and the different conceptualizations of loss of paid employment as dependent variables. Second, in the multivariate Cox regression analyses all factors with a level of significance of *p* < 0.05 in the previous univariate analyses were retained in the model, except for factors with high multicollinearity (i.e., Spearman correlation coefficient ≥0.70) and factors that violated the proportional hazards assumption.

Missing data were not imputed. See Supplementary Material Table S2a,b for frequency and proportion of missing data for key variables. All analyses were performed using R statistical software (version 1.1.463) and the package ‘survival’ was used for the Cox regression analyses [37,38].

## 3. Results

### 3.1. Characteristics of Cancer Survivor Sample and Reference Sample

Table 1 shows the baseline characteristics of the cancer survivor sample and the reference sample. Table 2 shows the baseline characteristics of the cancer survivor sample and the reference sample having paid work.

The descriptive statistics for age and sex are exactly the same for the cancer survivor sample and the reference sample, as these characteristics were used to select the matched reference sample. The average age was 55.4 years and 57% were male. The two samples were also comparable in terms of work-related characteristics and income. In the cancer survivor sample 6% had flexible working hours and in the reference sample 5% had flexible working hours. Further, 10% of the cancer survivor sample and 11% of the reference sample were self-employed.

Within the cancer survivor sample, 40% were in stage III at diagnosis, and 23%, 23% and 13% in stage I, II and IV, respectively. Surgery was the most common treatment (95%), followed by chemotherapy (51%), radiotherapy (28%) and targeted treatment (6%). There were no signs of multicollinearity (Supplementary Material Table S3). Therefore, we had no reason not to include any of the characteristics because of presence of multicollinearity.

### 3.2. Comparison between Cancer Survivor Sample and the Population-Based Reference Sample

**Figure 2:**
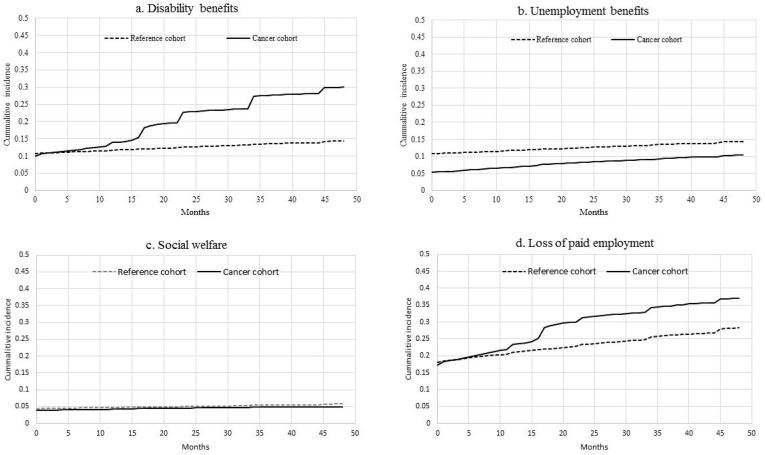
Cumulative incidences for (**a**) disability benefits, (**b**) unemployment benefits, (**c**) social welfare and (**d**) loss of paid employment for the cancer survivor cohort and the reference cohort.

A total of 67% of the cancer survivors had paid work the month before diagnosis compared to 70% of the controls (Table 1). Among the cancer survivors without paid employment at diagnosis, the most important income source was disability benefits (10%), followed by unemployment benefits (5%) and social welfare (4%). Among the reference sample without paid employment, the most important income source was also disability benefits (11%), followed by unemployment benefits (5%) and social welfare (4%) (Figure 2a–d).

In the four years of follow-up after diagnosis, disability benefits as the main income source increased from 10% to 30% for colorectal cancer survivors, whereas it remained rather stable in the reference sample (increase from 11% to 14%) (Figure 2a–d). In the univariate analysis, colorectal cancer survivors had an increased risk of having disability benefits as a main income source 0–2 years after diagnosis (hazard ratio (HR) 3.03, 95% CI 2.83–3.23) and 2–4 years after diagnosis (HR 4.41, 95% CI 3.91–4.99) (Supplementary Material Table S4a). Due to violation of the proportional hazards assumption, the multivariate analyses were only reported for 2–4 years after diagnosis. Overall, colorectal cancer survivors had a higher risk of having disability benefits as the main income source 2–4 years after diagnosis compared to a population based reference group (HR 4.41, 95% CI 3.90–4.98) (Table 3) in a multivariate model.

Having unemployment benefits as main income source increased for colorectal cancer survivors from 5% to 10% in four years, and for the controls from 5% to 13%. In the univariate analysis, colorectal cancer survivors had a lower risk of having unemployment benefits as the main income source (HR 0.73, 95% CI 0.66–0.79). All other independent variables in the univariate analyses either were not significant or they violated the proportional hazards assumption (Supplementary Material Table S4b). Thus, no multivariate model was built for unemployment benefits.

Having social welfare as the main income source remained rather stable for colorectal cancer survivors in four years (increase from 4% to 5%), and for the controls (increase from 4% to 6%) (Figure 2a–d). In the univariate analysis, there was no increased risk for colorectal cancer survivors having social welfare as the main income source compared to a population based reference group (Supplementary Material Table S4c).

Overall, loss of paid employment increased from 17% to 37% in four years for colorectal cancer survivors, and from 18% to 28% for the controls (Figure 2a–d). In the univariate analysis, colorectal cancer survivors had an increased risk of loss of paid employment 0–2 years after diagnosis (HR 1.60, 95% CI 1.52–1.68) and 2–4 years after diagnosis (HR 1.56, 95% CI 1.42–1.71) (Supplementary Material Table S4d). Due to violation of the proportional hazards assumption, the multivariate analyses are only reported for 2–4 years after diagnosis. Overall, colorectal cancer survivors had a higher risk of loss of paid employment 2–4 years after diagnosis compared to a population based reference group (HR 1.56, 95% CI 1.42–1.71) (Table 3) in a multivariate model.

### 3.3. Cancer Survivor Sample

#### 3.3.1. Disability Benefits

Step 1: univariate analyses

In the univariate analyses, we found that receiving chemotherapy (HR 1.72, 95% CI 1.54–1.91), radiotherapy (HR 1.19, 95% CI 1.07–1.32), targeted treatment (HR 2.44, 95% CI 2.09–2.84), having a higher cancer stage (IV vs. I) (HR 3.48, 95% CI 2.92–4.14) and older age (56–60 vs. 45–55) (HR 1.20, 95% CI 1.06–1.35) were associated with a higher probability of having disability benefits as the main income source 0–2 years after diagnosis, whereas receiving surgery (HR 0.42, 95% CI 0.35–0.49), being self-employed (HR 0.51, 95% CI 0.42–0.60) and having a higher income (>50 k vs. <500 €) (HR 0.24, 95% CI 0.17–0.33) were associated with a lower probability of having disability benefits as the main income source 0–2 years after diagnosis (Supplementary Material Table S5a).

We found in the univariate analyses that receiving chemotherapy (HR 2.23, 95% CI 1.86–2.68), radiotherapy (HR 1.61, 95% CI 1.35–1.92), targeted treatment (HR 2.12, 95% CI 1.59–2.82), having a higher cancer stage (IV vs. I) (HR 4.63, 95% CI 3.34–6.42) and being younger (45–55 vs. <45) (HR 1.37, 95% CI 1.04–1.81) were associated with a higher probability of having disability benefits as a main income source 2–4 years after diagnosis, whereas surgery (HR 0.53, 95% CI 0.38–0.74), being older (>60 vs. 45–55) (HR 0.65, 95% CI 0.51–0.83), being self-employed (HR 0.41, 95% CI 0.29–0.58) and higher income (>50 k vs. <500 €) (HR 0.30, 95% CI 0.16–0.53) were associated with a lower probability of having disability benefits as main income source 2–4 years after diagnosis (Supplementary Material Table S5a).

Step 2: multivariate analysis

Due to violation of the proportional hazards assumption, the multivariate analyses are only reported for 2–4 years after diagnosis. We found in the multivariate analyses that: receiving chemotherapy (HR 1.35, 95% CI 1.06–1.73), radiotherapy (HR 1.31, 95% CI 1.09–1.57) and having a higher cancer stage (IV vs. I) (HR 3.09, 95% CI 2.06–4.62) were associated with a higher probability of having disability benefits 2–4 years after diagnosis, whereas being older (>60 vs. 45–55) (HR 0.77, 95% CI 0.60–0.98) and being self-employed (HR 0.41, 95% CI 0.29–0.58) were associated with a lower probability of having disability as the main income source 2–4 years after diagnosis (Table 4).

#### 3.3.2. Unemployment Benefits

Step 1: univariate analyses

We found in the univariate analysis that only surgery (HR 1.99, 95% CI 1.19–3.32) was associated with a higher probability of having unemployment benefits as the main income source. Receiving chemotherapy (HR 0.69, 95% CI 0.59–0.82), targeted treatment (HR 0.51, 95% CI 0.32–0.81), having a higher cancer stage (IV vs. I) (HR 0.47, 95% CI 0.35–0.65), being self-employed (HR 0.44, 95% CI 0.32–0.60) and higher income (>50 k vs. <500 €) (HR 0.44, 95% CI 0.26–0.76) were associated with a lower probability of having unemployment benefits as the main income source (Supplementary Material Table S5b).

Step 2: multivariate analysis

We found in the multivariate analysis that having a higher cancer stage (IV vs. I) (HR 0.60, 95% CI 0.40–0.89) and being self-employed (HR 0.44, 95% CI 0.32–0.60) were associated with a lower probability of having unemployment benefits as the main income source (Table 4).

#### 3.3.3. Social Welfare

Step 1: univariate analyses

We found in the univariate analysis that having a higher cancer stage (IV vs. I) (HR 2.34, 95% CI 1.16–4.70) and being self-employed (HR 2.49, 95% CI 1.60–3.88) were associated with a higher probability of having social welfare as the main income source (Supplementary File 4c). Surgery (HR 0.49, 95% CI 0.25–0.98), higher age (>60 vs. 45–55) (HR 0.18, 95% CI 0.08–0.41) and higher income (>50 k vs. <500 €) (HR 0.01, 95% CI 0.00–0.04) were associated with a lower probability of having social welfare as the main income source (Supplementary Material Table S5c).

Step 2: multivariate analysis

We found no variables that were associated with a higher probability of having social welfare as main income source in the multivariate analysis. Only higher age (>60 vs. 45–55) (HR 0.21, 95% CI 0.09–0.46) was associated with a lower probability of having social welfare as the main income source (Table 4).

#### 3.3.4. Loss of Paid Employment

Step 1: univariate analyses

We found in the univariate analysis that receiving chemotherapy (HR 1.31, 95% CI 1.20–1.42), radiotherapy (HR 1.12, 95% CI 1.02–1.22), targeted treatment (HR 1.81, 95 CI 1.57–2.08), higher cancer stage (IV vs. I) (HR 2.03, 95% CI 1.77–2.33), higher age (56–60 vs. 45–55) (HR 1.16, 95% CI 1.04–1.28) and being self-employed (HR 1.28, 95% CI 1.14–1.43) were associated with a higher probability of loss of paid employment 0–2 years after diagnosis. Surgery (HR 0.51, 95% CI 0.44–0.60) and higher income (>50 k vs. <500 €) (HR 0.23, 95% CI 0.18–0.29) were associated with a lower probability of loss of paid employment 0–2 years after diagnosis (Supplementary Material Table S5d).

We found in the univariate analysis that receiving chemotherapy (HR 1.66, 95% CI 1.41–1.96), radiotherapy (HR 1.58, 95% CI 1.34–1.86), targeted treatment (HR 1.63, 95 CI 1.21–2.20), higher cancer stage (IV vs. I) (HR 2.55, 95% CI 1.94–3.37) and younger age (<45 vs. 45–55) (HR 1.31, 95% CI 1.01–1.70) were associated with a higher probability of loss of paid employment 2–4 years after diagnosis. Surgery (HR 0.56, 95% CI 0.41–0.77), higher age (>60 vs. 45–55) (HR 0.57, 95% CI 0.45–0.71) and higher income (>50 k vs. <500 €) (HR 0.44, 95% CI 0.25–0.77) were associated with a lower probability of loss of paid employment 2–4 years after diagnosis (Supplementary Material Table S5d).

Step 2: multivariate analysis

Due to violation of the proportional hazards assumption, the multivariate analyses are only reported for 2–4 years after diagnosis. We found in the multivariate analysis that radiotherapy (HR 1.37, 95% CI 1.15–1.63) and higher cancer stage (IV vs. I) (HR 1.89, 95% CI 1.33–2.70) were associated with a higher probability of having loss of paid employment (Table 4). Higher age (>60 vs. 45–55) (HR 0.64, 95% CI 0.51–0.81) was associated with a lower probability of having loss of paid employment.

## 4. Discussion

### 4.1. Interpretation of the Findings

We found that the percentage of colorectal cancer survivors that had paid employment at diagnosis is comparable to a population-based reference sample. Furthermore, we found that the colorectal cancer survivors had a 1.56 higher risk of loss of paid employment compared to a reference sample, mainly due to work disability. The overall loss of paid employment increased from 17% to 37% in four years for colorectal cancer survivors, and from 18% to 28% for the controls. This is in line with previous research that found a HR of 1.60 [36] and a relative risk (RR) of 1.44 [4] in samples of breast and gastrointestinal cancer survivors, respectively. In the Netherlands, employees who are (partly) sick-listed for two years may qualify for a work disability pension and receive up to 70% of their last income. Within these first two years of sick leave it is therefore unlikely to transition into social welfare. Additionally, employees may transition into unemployment when their employment contracts ends, but only when they are no longer sick-listed. It is, thus, not surprising that the overall risk of loss of paid employment through disability pension is higher than through unemployment and social welfare, and that the risk of loss of paid employment through unemployment is even lower among colorectal cancer survivors.

Remarkably, sex was not associated with any of the work-related outcomes. This is in line with previous research on colorectal cancer [23,39,40] but in contrast to the wealth of literature on other cancer types [41], cardiovascular events [42] and various chronic conditions [43]. In general, the association between sex and work-related outcomes is complex because of sex differences with regard to diagnosis, colorectal cancer screening [44], type of work and gender roles [45]. Further research is needed disentangling the different mechanisms related to sex and work-related outcomes.

In comparison with previous research on prognostic factors for colorectal cancer, we found in this study that receiving adjuvant treatment and higher cancer stage was associated with a higher risk of loss of paid employment [23,26,46]. Our finding that only radiotherapy remained statistically significant in the multivariate model indicates that it is important not to combine these forms of adjuvant treatment into one variable. Previous findings about associations with age are inconclusive. Some studies found that higher age was associated with loss of paid employment [23,24] others found the opposite [40], just like we did. Possible explanations for our finding may include that older colorectal cancer survivors left the workforce through another exit route than the three that we studied, i.e., early retirement, younger colorectal cancer survivors might have more difficulties with combining care for children at home with returning to work and/or a healthy worker effect.

We found a 2.49 higher risk for self-employed colorectal cancer survivors to enter social welfare 0–4 years after diagnosis in the univariate analysis. However, in the multivariate model the association is no longer statistically significant. We tested whether this was due to an interaction effect with age or stage but could not find any univocal trend (data not shown). As we also noticed that the 95% CI became rather large, we conclude that it would be recommended for further research to study in a larger sample whether this risk is actually due to being self-employed or interaction with other sociodemographic, cancer- or work-related variables. As the number of self-employed persons is increasing in Western countries and not much is known on the consequence of a cancer diagnosis on self-employment, this would be of added value [47].

### 4.2. Strengths and Limitations

The use of a population-based sample, including all colorectal cancer survivors, can be regarded as a strength of this study. A population-based sample is a non-selective sample by definition. This is an advantage in comparison to non-register-based studies that have to deal with lost to follow-up. However, register-based studies have limitations as well, such as limited factors that can be studied and absence of experience-based factors such as fatigue, self-assessed work ability and employer support, which are known to be prognostic factors for loss of paid employment among people with other cancer types (e.g., [20]). A limitation of this specific register-based study is that we have no information on subsequent cancer, level of education and other work-related characteristics besides the ones that we have studied. Previous research indicated that these factors are associated with loss of paid employment in cancer survivors as well. Thus, further research is needed in which register-based and self-reported studies are combined to better understand which colorectal cancer survivors are at high risk of loss of paid employment. Another limitation of our study is that inclusion criteria for the cancer survivor sample were not exactly the same as for the population-based reference sample. That is, the reference sample was not selected on being alive two years after baseline, whereas the cancer survivor sample was. However, the odds that someone dies within this two-year period are negligible, thus we assume that this has barely influenced our findings. Furthermore, living in the Netherlands with a known source of income was an inclusion criterion for the reference sample, whereas it was not for the sample of colorectal cancer survivors. Cancer patients are only registered in the NCR when they are treated in the Netherlands, and thus, most likely also have a known source of income in the Netherlands. Consequently, this has most likely not impacted our findings.

### 4.3. Recommendations for Further Research and Practice

Considering our results and the strengths and limitations of this study, we recommend first and foremost for practice that work supportive interventions for colorectal cancer survivors who are younger and who receive radiotherapy are developed and studied in terms of reach, compliance and (cost-)effectiveness. It is unclear whether this is also desirable for those diagnosed with a higher cancer stage as they may have a higher risk of dying within the first two years after diagnosis and thus may not have been included in the current analyses. Return-to-work interventions for colorectal cancer are scarce but previous research indicated that return-to-work interventions as part of cancer survivorship care can be delivered by a specialist cancer nurse and/or occupational physician specialized in cancer [48,49].

For research, we recommend that the advantages of both types of studies, i.e., studies based on register-data and studies based on self-reported data, are combined in one study. For instance, when self-reported prognostic factors and outcomes are individually matched to registers, we will be able to study the extent of the selection bias (e.g., [50]) and estimate its effect on register-based outcomes. Furthermore, we would have better understanding which patients are at high risk and on which factors we should intervene with supportive interventions. Additionally, in this study, based on theoretical grounding, we made a lot of choices in different stages of the research, among others with regard to the composition of the cancer survivor sample and the reference sample. Our cancer survivor sample was composed of colorectal cancer survivors who were alive 2 years after diagnosis as we focused on cancer survivorship care. Our reference sample was composed of people without a colorectal cancer diagnosis 2012–2016, who are not necessarily healthy and/or diagnosed with colorectal cancer beyond the study period or any other cancer during the whole period. In some other population-based studies, the reference population is composed of cancer free controls [51,52]. This may lead to an overestimation of the true effect of a colorectal cancer diagnosis on loss of paid employment as workers may have been diagnosed with other cancer types, which are also associated with a loss of paid employment [36]. Therefore, it would be recommended for further research, to repeat—in one sample—the same statistical analysis on different compositions of the cancer cohort and the reference cohort to have a clearer idea what the effect is of each decision.

## 5. Conclusions

In conclusion, we found that colorectal cancer survivors having paid work at their diagnosis had a higher risk of loss of employment up to 4 years after diagnosis compared to a reference population. Within the group of colorectal cancer survivors, those being younger, having a higher cancer stage and receiving radiotherapy, had a higher risk of loss of paid employment. These colorectal cancer survivors may benefit from work support as part of cancer survivorship care.

## Figures and Tables

**Table 1:** Characteristics of the cancer survivor cohort and the reference cohort.

		Cancer Survivor Cohort(*N* = 12,007)	Reference Cohort(*N* = 48,028)
Paid work one month before diagnosis (*N* (% yes))		8275 (69%)	32,143 (68%)
Age at time of colorectal cancer diagnosis (mean (±SD))		55.4 (±6.9)	55.4 (± 6.9) ^1^
Sex *N* (% male)		6844 (57%)	27,444 (57%) ^1^
Treatment *	Surgery	11,400 (95%)	NA
	Chemotherapy	6093 (51%)	
	Radiotherapy	3398 (28%)	
	Targeted	695 (6%)	
Stage at diagnosis	I	2800 (23%)	NA
	II	2762 (23%)	
	III	4860 (40%)	
	IV	1585 (13%)	
Flex work (*N* (% yes))		464 (6%)	1735 (5%)
Self-employed (*N* (% yes))		1242 (10%)	5118 (11%)
Income before diagnosis	<500	2755 (23%)	11,778 (25%)
	500–20 k	2262 (19%)	8785 (18%)
	20 k–35 k	2216 (19%)	8808 (19%)
	35 k–50 k	2166 (18%)	8207 (17%)
	>50 k	2535 (21%)	9934 (21%)

^1^. Exactly the same as the reference cohort was matched on age and sex. Percentages do not add up due to rounding. * Percentages equal to treatment/diagnosis.

**Table 2:** Characteristics of the cancer survivor cohort and the reference cohort having paid work.

		Cancer Survivor Cohort(*N* = 8275)	Reference Cohort(*N* = 32,143)
Age at diagnosis/baseline	<45	742 (9%)	3000 (9%)
	45–55	2673 (32%)	10,272 (32%)
	56–60	2447 (30%)	9645 (30%)
	>60	2413 (29%)	9226 (29%)
Sex *N* (% male)		5150 (62%)	19,906 (62%)
Treatment *	Surgery	7852 (95%)	NA
	Chemotherapy	4323 (52%)	
	Radiotherapy	2453 (30%)	
	Targeted	507 (6%)	
Stage at diagnosis	I	1835 (22%)	NA
	II	1900 (23%)	
	III	3402 (41%)	
	IV	1138 (14%)	
Flex work (*N* (% yes))		316 (6%)	1193 (6%)
Self-employed (*N* (% yes))		1242 (15%)	5118 (16%)
Income before diagnosis	<500	153 (2%)	597 (2%)
	500–20 k	1764 (21%)	6774 (21%)
	20 k–35 k	2071 (25%)	8170 (26%)
	35 k–50 k	2048 (25%)	7686 (24%)
	>50 k	2217 (27%)	8800 (27%)

* Percentages equal to treatment/diagnosis.

**Table 3:** Hazard ratios of work-related outcomes in multivariate models in the total cohort having paid work.

		Disability Benefits (2–4 Years) ^1^		Unemployment Benefits 0–4 Years ^2^		Social Welfare 0–4 Years ^3^		Loss of Paid Employment (2–4 Years) ^4^	
Number of Observations		*N* = 24,655		*N* = 40,418		*N* = 40,418		*N* = 22,865	
		HR (95 CI%)	*p*-Value	HR (95 CI%)	*p*-Value	HR (95 CI%)	*p*-Value	HR (95 CI%)	*p*-Value
Colorectal cancer	Colorectal cancer	4.41 (3.90, 4.98)	<0.001	0.73 (0.66,0.79)	<0.001	-	-	1.56 (1.42, 1.71)	<0.001
	No colorectal cancer *	Reference	-	Reference	-	-	-	Reference	-
Age at diagnosis/baseline	<45	1.16 (0.94, 1.43)	0.167	-	-	1.31 (1.00, 1.72)	0.048	1.21 (1.05, 1.39)	0.007
	45–55 *	Reference		-	-	Reference		Reference	
	56–60	1.02 (0.88, 1.19)	0.779	-	-	0.67 (0.53, 0.84)	0.001	1.00 (0.90, 1.10)	0.965
	>60	0.70 (0.59, 0.83)	<0.001	-	-	0.39 (9.29, 0.53)	<0.001	0.70 (0.62, 0.78)	<0.001
Sex	Male	-	-	-	-	0.59 (0.49, 0.71)	<0.001	-	-
	Female *	-	-	-	-	Reference	-	-	
Self-employed	Yes	0.42 (0.34, 0.54)	<0.001	-	-	1.37 (1.07, 1.75)	0.011	0.66 (0.57, 0.75)	<0.001
	No *	Reference		-	-	Reference	-	Reference	-

* Reference. ^1^. Proportional hazard assumption check *p*-value 0.11. Concordance index 0.69; ^2^. Proportional hazard assumption check *p*-value 0.36. Concordance index 0.53; ^3^. Proportional hazard assumption check *p*-value 0.001. Concordance index 0.58; ^4^. Proportional hazard assumption check *p*-value 0.31. Concordance index 0.64.

**Table 4:** Hazard ratios of work-related outcomes in multivariate model in the cancer survivor cohort having paid work.

		Disability Benefits (2–4 Years) ^1^		Unemployment Benefits (0–4 Years) ^2^		Social Welfare (0–4 Years) ^3^		Loss of Paid Employment (2–4 Years) ^4^	
Number of Observations		*N* = 4694		*N* = 8275		*N* = 8275		*N* = 4290	
		HR (95 CI%)	*p*-Value	HR (95 CI%)	*p*-Value	HR (95 CI%)	*p*-Value	HR (95 CI%)	*p*-Value
Surgery	Yes	0.91 (0.64, 1.31)	0.626	1.34 (0.77, 2.34)	0.300	0.51 (0.23, 1.12)	0.092	0.84 (0.60, 1.18)	0.310
	No *	Reference	-	-	-	-	-	-	-
Chemotherapy	Yes	1.35 (1.06, 1.73)	0.017	0.89 [0.70, 1.13)	0.345	-	-	1.22 (0.97, 1.53)	0.087
	No *	Reference	-	-	-	-	-	-	-
Radiotherapy	Yes	1.31 (1.09, 1.57)	0.004	-	-	-	-	1.37 (1.15, 1.63)	<0.001
	No *	Reference	-	-	-	-	-	-	-
Targeted	Yes	1.07 (0.73, 1.56)	0.731	0.78 [0.45, 1.38)	0.398	-	-	0.92 (0.63, 1.36)	0.684
	No *	Reference	-	-	-	-	-	-	-
Cancer stage	I *	Reference	-	Reference	-	Reference	-	Reference	
	II	1.67 (1.19, 2.34)	0.003	0.86 [0.68, 1.08)	0.188	1.75 (0.89, 3.44)	0.106	1.20 (0.92, 1.58)	0.186
	III	2.03 (1.42, 2.91)	<0.001	0.72 [0.54, 0.96)	0.026	1.32 (0.70, 2.48)	0.399	1.26 (0.92, 1.71)	0.129
	IV	3.09 (2.06, 4.62)	<0.001	0.60 [0.40, 0.89)	0.012	1.75 (0.82, 3.74)	0.150	1.89 (1.33, 2.70)	<0.001
Age at diagnosis	<45	1.32 (1.00, 1.74)	0.053	-	-	0.74 (0.39, 1.43)	0.377	1.30 (1.00, 1.69)	0.054
	45–55 *	Reference	-	-	-	-	Reference	-	Reference
	56–60	1.06 (0.86, 1.30]	0.605	-	-	0.62 (0.38, 1.00)	0.052	1.04 (0.85, 1.26)	0.715
	>60	0.77 (0.60, 0.98]	0.035	-	-	0.21 (0.09, 0.46)	<0.001	0.64 (0.51, 0.81)	<0.001
Self-employed	Yes	0.41 (0.29, 0.58]	<0.001	0.44 [0.32, 0.60)	<0.001	1.28 (0.79, 2.08)	0.317	-	-
	No *	Reference	-	Reference		Reference	-	-	-

* Reference. ^1^. Proportional hazard assumption check *p*-value 0.19. Concordance index 0.68; ^2^. Proportional hazard assumption check *p*-value 0.01. Concordance index 0.58; ^3^. Proportional hazard assumption check *p*-value 0.04. Concordance index 0.63; ^4^. Proportional hazard assumption check *p*-value 0.18. Concordance index 0.88.

## Data Availability

Restrictions apply to the availability of these data. Data was obtained from the Netherlands Cancer Registry and Statistics Netherlands. Under certain conditions, the data are accessible for statistical and scientific research with the permission of these third parties.

## Supplementary Materials

The following are available online at https://www.mdpi.com/article/10.3390/cancers13122868/s1, Table S1: detailed explanation of each variable, Table S2: correlation matrix showing the correlation of all variables of the multivariate model with each other, Table S3a: factors potentially associated with work disability benefits tested in univariate model cancer cohort and reference cohort, Table S3b: factors potentially associated with unemployment benefits tested in univariate model cancer cohort and reference cohort, Table S3c: factors potentially associated with social welfare tested in univariate model cancer cohort and reference cohort, Table S3d: factors potentially associated with loss of paid employment tested in univariate model cancer cohort and reference cohort, Table S4a: factors associated with disability benefits tested in univariate model cancer cohort, Table S4b: factors associated with unemployment benefits tested in univariate model cancer cohort, Table S4c: factors associated with social welfare tested in univariate model cancer cohort, Table S4d: factors associated with loss of paid employment tested in univariate model cancer cohort, Table S5a: factors associated with disability benefits tested in univariate model cancer cohort, Table S5b: factors associated with unemployment benefits tested in univariate model cancer cohort, Table S5c: factors associated with social welfare tested in univariate model cancer cohort, Table S5d: factors associated with loss of paid employment tested in univariate model cancer cohort.
