# Supplementary material for: Loss of Paid Employment up to 4 Years after Colorectal Cancer Diagnosis—A Nationwide Register-Based Study with a Population-Based Reference Group
Source: Cancers (Basel). 2021 Jun 8;13(12):2868. doi: 10.3390/cancers13122868 (PMC8229293; doi:10.3390/cancers13122868)
Supplement: Supplementary file 1 [file cancers-13-02868-s001.zip › cancers-1248198-supplementary.pdf]

cancers

MDPI

Supplementary Materials: Loss of Paid Employment up to 4 Years after Colorectal Cancer Diagnosis-A Nationwide Register-Based Study with a Population-Based Reference Group

Astrid de Wind, Sietske J. Tamminga, Claudia A.G. Bony, Maren Diether, Martijn Ludwig, Miranda J. Velthuis, Saskia F.A. Duijts and Angela G.E.M. de Boer

Table S1. Detailed explanation of each variable.

Registry	Data file	Variable used	Definition of variable	Categories within variable	Definition of category	Detailed explanation of each category
Statistics Netherlands	SECMBUS	SECM	Most important income source	XKOPPELZIEKTEAOSECM	Receiver disability benefits in month in question (yes/no)	For employees/ temporary agency workers a disability pension can either be applied for when being on sick leave for 2 years or earlier in the sick leave process when recovery is considered very unlikely. An employee qualifies for disability pension when recovery is considered very unlikely or when someone is considered unable to earn more than 35% of his/her previous salary given his/her limitations. Self-employed are not obliged to be insured against work disability in the Netherlands.
				XKOPPELWERKLUITKSECM	Receiver unemployment benefits in month in question (yes/no)	When an employee/temporary agency worker losses his/her job he/she can apply for unemployment benefits. The duration depends on someone's employment history. Self-employed are not obliged to be insured against unemployment in the Netherlands.
				XKOPPELBIJSTANDSECM	Receiver social welfare in month in question (yes/no)	When unemployment benefits are expired or when someone does not qualify for unemployment benefits someone can apply for social welfare.
				XKOPPELWERKNSECM	Working as employee (yes/no)	Having a job as an employee in the Netherlands or income from paid work abroad (excluding director-major shareholder)
				XKOPPELZELFSTSECM	Working as self-employed person (yes/no)	Having profit from a business.
	SPOLISBUS	SARBEIDSRELATIE	Categorization of jobs based on agreement in the employment contract on the flexibility of working hours	Fixed<br>Flexible	Flex work (yes/no)	Fixed employment relationship includes jobs with fixed working hours. Flexible employment relationship includes on-call and temporary agency workers.

	INPATAB	INPPERSINK	Personal gross income from paid work, capital, disability benefits, unemployment benefits, and/or social welfare			Continuous variable, we made the following categories for this study <500, 500-20k, 20k-35k, 35k-50k >50k
	GBAPERSOONTAB	GBAGESLACHT	Sex	Male<br>Female		In the case of change of sex only the last current sex is registered. In the case sex is unknown a person is registered as female.
		GBAGEBOORTEJAAR<br>GBAGEBOORTEMAAND	Birth year<br>Birth month			Age at time of diagnosis (or reference year) is calculated as continuous variable. We used the following age group strata: <45, 45-54, 55-59 and 60-62 years.
Netherlands Cancer Registry			Cancer diagnosis	Topog		C180 Coecum<br>C181 Appendix<br>C182 Colon ascendens<br>C183 Flexura hepatica<br>C184 Colon transversum<br>C185 Flexura lienalis<br>C186 Colon descendens<br>C187 Colon sigmoideum<br>C188 Colon overlapping<br>C189 Colon NNO<br>C199 Rectosigmoid<br>C209 Rectum
			Cancer treatment	Indchorg<br>Indchlok<br>Indchov	Surgery (yes/no)	
				Indchemo	Received chemotherapy (yes/no)	
				Indrt	Received radiotherapy (yes/no)	
				Indtarget	Received targeted therapy (yes/no)	
			Cancer stage	Stadiumc	I, II, III or IV	Cancer stage based on TNM classification 7th edition

Table S2. a: frequency and proportion of missing data for key variables in total sample.

	Disability benefits (0–4 years) (*N* = 40,418)	Unemployment benefits (0–4 years) (*N* = 40,418)	Social welfare (0–4 years) (*N* = 40,418)	Loss of paid employment (0–4 years) (*N* = 40,418)
Age	0	0	0	0
Sex	0	0	0	0
Flex work	13,705 (34%)	13,705 (34%)	13,705 (34%)	13,705 (34%)
Self-employed	0	0	0	0
Income	138 (0.3%)	138 (0.3%)	134 (0.3%)	138 (0.3%)

Table S2. b: frequency and proportion of missing data for key variables in colorectal cancer sample.

	Disability benefits (0-4 years) (*N* = 8,275)	Unemployment benefits (0-4 years) (*N* = 8,275)	Social welfare (0-4 years) (*N* = 8,275)	Loss of paid employment (0-4 years) (*N* = 8,275)
Age	0	0	0	0
Sex	0	0	0	0
Flex work	2,858 (35%)	2,858 (35%)	2,858 (35%)	2,858 (35%)
Self-employed	0	0	0	0
Income	22 (0.3%)	16 (0.2%)	22 (0.3%)	13 (0.2%)
Treatment	0	0	0	0
Stage at diagnosis	0	0	0	0

Table S3. correlation matrix showing the correlation of all variables of the multivariate model with each other.

	Age <45	Age 45-55	Age 55-60	Age 60+	Self-employed	Income <500	Income 500-20k	Income 20k-35k	Income 35k-50k	Income >50k	Cancer stage I	Cancer stage II	Cancer stage III	Cancer stage IV	Sex	Chemo -therapy	Surgery	Targeted	Radio -therapy
**Age <45**	1.00	-0.19	-0.19	-0.21	0.01	-0.05	0.02	0.04	-0.01	-0.01	-0.05	0.00	0.02	0.04	-0.05	0.06	-0.01	0.03	0.01
**Age 45-55**	-0.19	1.00	-0.41	-0.46	0.04	-0.11	0.03	0.04	0.02	0.03	-0.09	-0.01	0.06	0.04	-0.04	0.09	-0.02	0.04	0.07
**Age 55-60**	-0.19	-0.41	1.00	-0.46	-0.02	-0.01	0.01	-0.02	0.01	0.02	-0.03	0.02	0.01	0.00	0.00	0.00	-0.01	-0.02	0.04
**Age 60+**	-0.21	-0.46	-0.46	1.00	-0.03	0.15	-0.05	-0.05	-0.02	-0.03	0.15	-0.01	-0.07	-0.07	0.06	-0.12	0.04	-0.04	-0.10
**Self-employed**	0.01	0.04	-0.02	-0.03	1.00	-0.12	0.09	0.03	-0.01	0.03	0.00	0.00	0.00	0.00	0.09	0.00	-0.02	0.00	0.03
**Income <500**	-0.05	-0.11	-0.01	0.15	-0.12	1.00	-0.26	-0.26	-0.26	-0.28	0.04	0.01	-0.02	-0.03	-0.19	-0.05	0.02	-0.03	-0.05
**Income 500-20k**	0.02	0.03	0.01	-0.05	0.09	-0.26	1.00	-0.23	-0.23	-0.25	0.00	0.00	-0.01	0.01	-0.25	-0.01	0.01	-0.02	-0.01
**Income 20k-35k**	0.04	0.04	-0.02	-0.05	0.03	-0.26	-0.23	1.00	-0.22	-0.25	-0.02	0.01	0.01	0.00	-0.05	0.01	0.00	0.01	0.01
**Income 35k-50k**	-0.01	0.02	0.01	-0.02	-0.01	-0.26	-0.23	-0.22	1.00	-0.24	-0.02	-0.01	0.02	0.00	0.18	0.02	-0.01	0.01	0.03
**Income >50k**	-0.01	0.03	0.02	-0.03	0.03	-0.28	-0.25	-0.25	-0.24	1.00	-0.01	-0.01	0.00	0.02	0.31	0.02	-0.02	0.03	0.01
**Cancer stage I**	-0.05	-0.09	-0.03	0.15	0.00	0.04	0.00	-0.02	-0.02	-0.01	1.00	-0.30	-0.45	-0.22	0.00	-0.50	0.11	-0.14	-0.25
**Cancer stage II**	0.00	-0.01	0.02	-0.01	0.00	0.01	0.00	0.01	-0.01	-0.01	-0.30	1.00	-0.45	-0.21	-0.02	-0.29	0.10	-0.13	-0.10
**Cancer stage III**	0.02	0.06	0.01	-0.07	0.00	-0.02	-0.01	0.01	0.02	0.00	-0.45	-0.45	1.00	-0.32	0.03	0.47	0.08	-0.18	0.30
**Cancer stage IV**	0.04	0.04	0.00	-0.07	0.00	-0.03	0.01	0.00	0.00	0.02	-0.22	-0.21	-0.32	1.00	-0.01	0.29	-0.38	0.59	0.00
**Sex**	-0.05	-0.04	0.00	0.06	0.09	-0.19	-0.25	-0.05	0.18	0.31	0.00	-0.02	0.03	-0.01	1.00	-0.01	-0.02	0.00	0.08
**Chemotherapy**	0.06	0.09	0.00	-0.12	0.00	-0.05	-0.01	0.01	0.02	0.02	-0.50	-0.29	0.47	0.29	-0.01	1.00	-0.36	0.30	0.11
**Surgery**	-0.01	-0.02	-0.01	0.04	-0.02	0.02	0.01	0.00	-0.01	-0.02	0.11	0.10	0.08	-0.38	-0.02	-0.36	1.00	-0.41	-0.15
**Targeted**	0.03	0.04	-0.02	-0.04	0.00	-0.03	-0.02	0.01	0.01	0.03	-0.14	-0.13	-0.18	0.59	0.00	0.30	-0.41	1.00	-0.02
**Radiotherapy**	0.01	0.07	0.04	-0.10	0.03	-0.05	-0.01	0.01	0.03	0.01	-0.25	-0.10	0.30	0.00	0.08	0.11	-0.15	-0.02	1.00

Table S4. a: factors potentially associated with work disability benefits tested in univariate model cancer cohort and reference cohort.

		**Disability benefits 0-4 years**		**Disability benefits (0-2 years)**			**Disability benefits (2-4 years)**		
		**No (%)**	**Yes (%)**	**HR (95 CI%)**	***P*-Value**	**Proportional Hazard assumption check *P*-Value**	**HR (95 CI%)**	***P*-Value**	**Proportional Hazard assumption check *P*-Value**
Colorectal cancer	Cancer survivor cohort	81%	19%	3.03 [2.83,3.23]	<0.001	<0.01	4.41 [3.91,4.99]	<0.001	0.18
	Reference cohort*	94%	6%	Reference			Reference		
Age at diagnosis/baseline	<45	93%	7%	0.86 [0.75,0.98]	0.024	<0.001	1.13 [0.92,1.40]	0.243	0.80
	45-55*	92%	8%	Reference			Reference		
	55-60	90%	10%	1.30 [1.20,1.41]	<0.001		1.02 [0.88,1.18]	0.812	
	60+	91%	9%	1.48 [1.36,1.61]	<0.001		0.72 [0.60, 0.85]	<0.001	
Sex	Male*	91%	9%	1.11 [1.04, 1.19]	0.002	<0.001	0.99 [0.87, 1.12]	0.844	0.45
	Female	91%	9%	Reference			Reference		
Flex work	Yes	91%	9%	0.97 [0.81,1.16]	0.731	0.26	1.04 [0.75,1.44]	0.808	0.85
	No*	91%	9%	Reference			Reference		
Self-employed	Yes	94%	6%	0.64 [0.58,0.71]	<0.001	<0.001	0.41 [0.33,0.52]	<0.001	0.02
	No*	90%	10%	Reference			Reference		
Income before diagnosis	<500*	84%	16%	Reference		<0.001	Reference		<0.001
	500-20k	85%	15%	0.95 [0.78, 1.14]	0.574		1.37 [0.86, 2.18]	0.183	
	20k-35k	88%	12%	0.71 [0.59, 0.86]	<0.001		1.25 [0.79, 1.98]	0.349	
	35k-50k	94%	6%	0.36 [0.29, 0.43]	<0.001		0.74 [0.46, 1.19]	0.215	
	>50k	97%	3%	0.17 [0.14, 0.21]	<0.001		0.40 [0.25, 0.65]	<0.001	

Table S4. b: factors potentially associated with unemployment benefits tested in univariate model cancer cohort and reference cohort.

		Unemployment benefits 0-4 years				
		**No (%)**	**Yes (%)**	**HR (95 CI%)**	***P*-Value**	**Proportional Hazard assumption check *P*-Value**
Colorectal cancer	Cancer survivor cohort	93%	7%	0.73 [0.66, 0.79]	<0.001	0.36
	Reference cohort*	90%	10%	Reference		
Age at diagnosis/baseline	<45	89%	11%	1.09 [0.97,1.22]	0.136	0.24
	45-55*	90%	10%	Reference		
	55-60	90%	10%	1.02 [0.95,1.11]	0.560	
	60+	93%	7%	1.02 [0.95,1.11]	0.699	
Sex	Male*	91%	9%	0.94 [0.88,1.00]	0.052	0.001
	Female	90%	10%	Reference		
Flex work	Yes	91%	9%	0.92 [0.78, 1.10]	0.380	0.39
	No*	91%	9%	Reference		
Self-employed	Yes	96%	4%	0.43 [0.38,0.49]	<0.001	<0.001
	No*	90%	10%	Reference		
Income before diagnosis	<500*	86%	14%	Reference		<0.001
	500-20k	86%	14%	0.96 [0.79,1.17]	0.701	
	20k-35k	90%	10%	0.66 [0.54,0.80]	<0.001	
	35k-50k	94%	6%	0.40 [0.33,0.50]	<0.001	
	>50k	94%	6%	0.38 [0.31,0.47]	<0.001	

Table S4. c: factors potentially associated with social welfare tested in univariate model cancer cohort and reference cohort.

		Social welfare 0-4 years				
		**No (%)**	**Yes (%)**	**HR (95 CI%)**	***P*-Value**	**Proportional Hazard assumption check *P*-Value**
Colorectal cancer	Cancer survivor cohort	99%	1%	1.11 [0.88,1.39]	0.393	0.91
	Reference cohort*	99%	1%	Reference		
Age at diagnosis/baseline	<45	98%	2%	1.34 [1.03,1.76]	0.031	0.01
	45-55*	99%	1%	Reference		
	55-60	99%	1%	0.65 [0.51,0.82]	<0.001	
	60+	100%	0%	0.37 [0.27,0.50]	<0.001	
Sex	Male*	99%	1%	0.55 [0.46,0.67]	<0.001	0.02
	Female	99%	1%	Reference		
Flex work	Yes	99%	1%	1.42 [0.92,2.20]	0.113	0.67
	No*	99%	1%	Reference		
Self-employed	Yes	99%	1%	1.29 [1.01,1.64]	0.039	0.05
	No*	99%	1%	Reference		
Income before diagnosis	<500*	93%	7%	Reference		<0.001
	500-20k	96%	4%	0.45 [0.34,0.59]	<0.001	
	20k-35k	100%	0%	0.04 [0.03,0.07]	<0.001	
	35k-50k	100%	0%	0.02 [0.01,0.03]	<0.001	
	>50k	100%	0%	0.01 [0.00,0.01]	<0.001	

Table S4. d: factors potentially associated with loss of paid employment tested in univariate model cancer cohort and reference cohort.

		Loss of paid employment (0–4 years)		Loss of paid employment (0-2 years)			Loss of paid employment (2–4 years)		
		No (%)	Yes (%)	HR (95 CI%)	*P*-Value	Proportional Hazard assumption check P-Value	HR (95 CI%)	*p*-Value	Proportional Hazard assumption check *P*-Value
Colorectal cancer	Cancer survivor cohort	73%	27%	1.60 [1.52,1.68]	<0.001	<0.001	1.56 [1.42,1.71]	<0.001	0.01
	Reference cohort*	83%	17%	Reference			Reference		
Age at diagnosis/baseline	<45	81%	19%	1.01 [0.93,1.10]	0.737	<0.001	1.21 [1.05,1.39]	0.007	0.31
	45-55*	81%	19%	Reference			Reference		
	55-60	79%	21%	1.18 [1.11,1.25]	<0.001		1.00 [0.90,1.11]	0.987	
	60+	82%	18%	1.28 [1.20,1.35]	<0.001		0.70 [0.63,0.79]	<0.001	
Sex	Male	80%	20%	1.08 [1.03,1.13]	0.001	<0.001	1.01 [0.93,1.10]	0.847	0.50
	Female*	81%	19%	Reference			Reference		
Flex work	Yes	81%	19%	0.98 [0.87,1.11]	0.793	0.73	1.03 [0.83,1.28]	0.789	0.92
	No*	81%	19%	Reference			Reference		
Self-employed	Yes	82%	18%	0.95 [0.90,1.02]	0.142	<0.001	0.56 [0.57,0.74]	<0.001	<0.001
	No*	81%	19%	Reference			Reference		
Income before diagnosis	<500*	61%	39%	Reference		<0.001	Reference		<0.001
	500-20k	68%	32%	0.72 [0.64,0.81]	<0.001		1.01 [0.74,1.38]	0.949	
	20k-35k	78%	22%	0.45 [0.40,0.51]	<0.001		0.88 [0.65,1.21]	0.432	
	35k-50k	87%	13%	0.26 [0.23,0.30]	<0.001		0.60 [0.44,0.82]	0.001	
	>50k	89%	11%	0.21 [0.19,0.24]	<0.001		0.57 [0.42,0.78]	<0.001	

Table S5. a: factors associated with disability benefits tested in univariate model cancer cohort.

		Disability benefits (0-2)			Disability benefits (2-4)		
		**HR (95 CI%)**	***P*-Value**	**Proportional Hazard assumption check *P*-Value**	**HR (95 CI%)**	***P*-Value**	**Proportional Hazard assumption check *P*-Value**
Stage	1	Reference		<0.001	Reference		0.32
	2	1.26 [1.04,1.53]	0.016		1.94 [1.39, 2.70]	<0.001	
	3	1.81 [1.54,2.13]	<0.001		3.04 [2.27,4.08]	<0.001	
	4	3.48 [2.92,4.14]	<0.001		4.63 [3.34, 6.42]	<0.001	
Chemotherapy		1.72 [1.54, 1.91]	<0.001	<0.001	2.23 [1.86,2.68]	<0.001	0.16
Radiotherapy		1.19 [1.07,1.32]	0.001	<0.001	1.61 [1.35,1.92]	<0.001	0.09
Surgery		0.42 [0.35,0.49]	<0.001	<0.001	0.53 [0.38,0.74]	<0.001	0.29
Targeted therapy		2.44 [2.09,2.84]	<0.001	<0.001	2.12 [1.59,2.82]	<0.001	0.26
Age at diagnosis	<45	1.01 [0.84,1.22]	0.887	0.13	1.37 [1.04,1.81]	0.027	0.43
	45-55*	Reference			Reference		
	55-60	1.20 [1.06,1.35]	0.004		0.98 [0.80,1.21]	0.852	
	60+	1.14 [0.99,1.30]	0.064		0.65 [0.51,0.83]	<0.001	
Sex	Male*	0.95 [0.85,1.05]	0.296	<0.001	0.89 [0.74,1.05]	0.168	0.52
	Female	Reference			Reference		
Flex work	Yes	1.01 [0.78,1.31]	0.924	0.35	1.25 [0.82, 1.90]	0.305	0.71

	No*	Reference			Reference		
Self-employed	Yes	0.51 [0.42,0.60]	<0.001	<0.001	0.41 [0.29,0.58]	<0.001	0.05
	No*	Reference			Reference		
Income before diagnosis	<500*	Reference		<0.001	Reference		<0.001
	500-20k	0.89 [0.66, 1.21]	0.466		0.96 [0.54, 1.68]	0.876	
	20k-35k	0.74 [0.55, 1.01]	0.054		0.87 [0.50, 1.53]	0.634	
	35k-50k	0.44 [0.32, 0.61]	<0.001		0.52 [0.29, 0.92]	0.025	
	>50k	0.24 [0.17, 0.33]	<0.001		0.30 [0.16, 0.53]	<0.001	

Table S5. b: factors associated with unemployment benefits tested in univariate model cancer cohort.

		Unemployment benefits (0-4 years)		
		**HR (95 CI%)**	***P*-Value**	**Proportional Hazard assumption check *P*-Value**
Surgery		1.99 [1.19,3.32]	0.009	0.02
Chemotherapy		0.69 [0.59,0.82]	<0.001	0.71
Radiotherapy		0.90 [0.76,1.08]	0.261	0.77
Targeted		0.51 [0.32,0.81]	0.005	0.002
Cancer stage	I	Reference		0.04
	II	0.85 [0.68,1.06]	0.138	
	III	0.66 [0.54,0.81]	<0.011	
	IV	0.47 [0.35,0.65]	<0.001	
Age at diagnosis	<45	1.11 [0.84,1.48]	0.457	0.64
	45-55*	Reference		
	55-60	1.08 [0.89,1.32]	0.432	
	60+	1.02 [0.82,1.27]	0.874	
Sex	Male*	0.94 [0.79,1.11]	0.455	0.71
	Female	Reference		
Flex work	Yes	0.89 [0.57,1.38]	0.595	0.35
	No*	Reference		
Self-employed	Yes	0.44 [0.32,0.60]	<0.001	0.03
	No*	Reference		
Income before diagnosis	<500*	Reference		<0.001
	500-20k	1.22 [0.72,2.06]	0.454	
	20k-35k	0.72 [0.42,1.22]	0.216	
	35k-50k	0.44 [0.25,0.75]	0.003	
	>50k	0.44 [0.26,0.76]	0.003	

Table S5. c: factors associated with social welfare tested in univariate model cancer cohort.

		Social welfare		
		**HR (95 CI%)**	***P*-Value**	**Proportional Hazard assumption check *P*-Value**
Surgery		0.49 [0.25,0.98]	0.045	0.66
Chemotherapy		1.31 [0.86,1.98]	0.204	0.24
Radiotherapy		0.80 [0.51,1.27]	0.354	0.29
Targeted		0.88 [0.36,2.17]	0.786	0.17
Cancer stage	I	Reference		0.80
	II	1.65 [0.84,3.24]	0.146	
	III	1.39 [0.74,2.63]	0.305	
	IV	2.34 [1.16,4.70]	0.017	
Age at diagnosis	<45	0.78 [0.40,1.49]	0.449	0.68
	45-55*	Reference		
	55-60	0.56 [0.35,0.91]	0.019	
	60+	0.18 [0.08,0.41]	<0.001	
Sex	Male*	0.69 [0.46,1.03]	0.069	0.56
	Female	Reference		
Flex work	Yes	0.85 [0.26,2.70]	0.777	0.80
	No*	Reference		
Self-employed	Yes	2.49 [1.60,3.88]	<0.001	0.15
	No*	Reference		
Income before diagnosis	<500*	Reference		0.01
	500-20k	0.30 [0.17,0.52]	<0.001	
	20k-35k	0.04 [0.02,0.09]	<0.001	
	35k-50k	0.02 [0.01,0.05]	<0.001	
	>50k	0.01 [0.00,0.04]	<0.001	

Table S5. d: factors associated with loss of paid employment tested in univariate model cancer cohort.

		Loss of paid employment (0-2 years)			Loss of paid employment (2-4 years)		
		HR (95 CI%)	*P*-Value	Proportional Hazard assumption check *P*-Value	HR (95 CI%)	P-Value	Proportional Hazard assumption check *P*-Value
Stage	1	Reference		<0.001	Reference		0.08
	2	1.00 [0.87,1.15]	0.964		1.39 [1.07, 1.81]	0.015	
	3	1.22 [1.09,1.38]	0.001		1.80 [1.42, 2.27]	<0.001	
	4	2.03 [1.77,2.33]	<0.001		2.55 [1.94, 3.37]	<0.001	
Chemotherapy		1.31 [1.20,1.42]	<0.001	<0.001	1.66 [1.41, 1.96]	<0.001	0.19
Radiotherapy		1.12 [1.02,1.22]	0.015	0.006	1.58 [1.34, 1.86]	<0.001	0.14
Surgery		0.51 [0.44,0.60]	<0.001	<0.001	0.56 [0.41, 0.77]	<0.001	0.55
Targeted therapy		1.81 [1.57,2.08]	<0.001	<0.001	1.63 [1.21, 2.20]	0.001	0.001
Age at diagnosis	<45	1.07 [0.92,1.25]	0.365	<0.001	1.31 [1.01,1.70]	0.046	0.18
	45-55*	Reference			Reference		
	55-60	1.16 [1.04,1.28]	0.006		0.98 [0.81, 1.19]	0.856	
	60+	1.10 [0.98,1.23]	0.091		0.57 [0.45, 0.71]	<0.001	
Sex	Male*	1.07 [0.98,1.17]	0.112	<0.001	1.07 [0.90,1.26]	0.455	0.90
	Female	Reference			Reference		
Flex work	Yes	1.01 [0.81,1.26]	0.915	0.49	1.15 [0.77, 1.72]	0.507	0.94

	No*	Reference			Reference		
Self-employed	Yes	1.28 [1.14,1.43]	<0.001	<0.001	1.00 [0.79,1.26]	0.990	<0.001
	No*	Reference			Reference		
Income before diagnosis	<500*	Reference		<0.001	Reference		<0.001
	500-20k	0.66 [0.52,0.83]	<0.001		0.81 [0.46,1.43]	0.475	
	20k-35k	0.46 [0.36,0.58]	<0.001		0.74 [0.42,1.30]	0.298	
	35k-50k	0.29 [0.23,0.38]	<0.001		0.49 [0.28,0.87]	0.015	
	>50k	0.23 [0.18,0.29]	<0.001		0.44 [0.25,0.77]	0.004	
